# Platelet-rich plasma does not influence magnetic resonance imaging findings of the gluteus muscles after total hip arthroplasty through the Hardinge approach

**DOI:** 10.1007/s00256-025-04876-8

**Published:** 2025-01-27

**Authors:** Anette Nieminen, Janne Nurminen, Anni Aavikko, Jani Puhakka, Jussi Haapala, Hannes Keemu, Juha Kukkonen, Ari Alho, Panu Uusalo, Keijo Mäkelä, Jussi Kosola

**Affiliations:** 1 Department of Orthopedics and Traumatology, Turku University Hospital, University of Turku, Luolavuorentie 2, 20700 Turku, Finland; 2 Department of Radiology, Turku University Hospital, University of Turku, Turku, Finland; 3 Department of Orthopedics and Traumatology, Helsinki University Hospital, University of Helsinki, Helsinki, Finland; 4 Department of Surgery, Satakunta Central Hospital, Pori, Finland; 5 Department of Anaesthesiology and Intensive Care, University of Turku, Turku, Finland; 6 Division of Perioperative Services, Intensive Care and Pain Medicine, Turku University Hospital, Turku, Finland; 7 Department of Orthopedics and Traumatology, Kanta-Häme Central Hospital, Hämeenlinna, Finland

**Keywords:** MRI, Total hip arthroplasty, Platelet-rich plasma

## Abstract

**Objective:**

Total hip arthroplasty through the Hardinge approach damages the hip abductor muscles. MRI can be used to assess adverse postoperative events. In this prospective randomized controlled trial, we evaluated MRI findings and whether platelet-rich plasma affected postoperative healing of the gluteal muscles (gluteus medius and minimus).

**Materials and methods:**

Forty patients with hip osteoarthritis requiring treatment with total hip arthroplasty, aged between 60 and 76 years, were included. Patients were randomized into two groups: 19 patients in the platelet-rich plasma group and 21 in the placebo group. Platelet-rich plasma or placebo was injected into the gluteus medius tendon incision line during closure. Postoperative hip MRI and plain radiographs were taken 3 and 12 months after surgery.

**Results:**

MRI showed fatty atrophy of the gluteal muscles in all 40 patients (100%), gluteal muscle tear in 11 patients (28%), and atrophy in 16 patients (40%) at both 3 and 12 months postoperatively. Fluid collections related to the operated hip joint were seen in 18 patients (45%) at 3 months and 13 patients (33%) at 12 months, heterotopic ossification formation in nine patients (23%) at 3 months, and 12 patients (31%) at 12 months. There were no significant differences in imaging findings between the two groups.

**Conclusion:**

MRI can be a valuable tool for evaluating postoperative healing after total hip arthroplasty. Fatty atrophy of the gluteal muscles was a common finding. Platelet-rich plasma injection into the gluteus medius tendon did not improve healing detected by MRI.

## Introduction

Total hip arthroplasty (THA) is a common orthopedic procedure worldwide [1]. One of the most used THA approaches is the modified Hardinge approach [2–4]. The disadvantage of the Hardinge approach is splitting the gluteus medius and minimus hip abductor muscles, which may cause weakness of hip abduction. There is also a risk of injury to the superior gluteal nerve [5]. The clinical outcomes of gluteal muscle damage are limping, positive Trendelenburg sign, and lateral hip pain [6]. The main advantage of the Hardinge approach is hip stability; the dislocation revision risk with the posterior approach is three times that of the Hardinge approach [7].

While plain radiograph imaging remains the first-line modality for postoperative hip imaging, magnetic resonance imaging (MRI) stands out due to its superior soft tissue demarcation. MRI is also the best imaging modality for assessing the periarticular structures. It is a useful tool for diagnosing tendon tears, muscle atrophy, fatty atrophy, fluid collections, heterotopic ossification, periprosthetic stress fractures, and osteolysis without using ionizing radiation [8–10]. A symptomatic patient after THA may sometimes benefit from an MRI. Pfirmann et al. [11] demonstrated that symptomatic patients had significantly more abductor tendon defects and fatty atrophy of the gluteus medius muscle after THA compared to asymptomatic patients.

Platelet-rich plasma (PRP) is an autologous platelet plasma concentrate made from the patient’s own blood by centrifugation. It contains high levels of growth factors that are essential for healing processes [12]. PRP is a safe treatment and has been widely used to treat tendon injuries and tendinopathies [13]. However, the efficacy of PRP on tendon healing is still unclear.

Previous studies have been published on the effects of PRP on tendon healing, mostly in rotator cuff or Achilles tendon injuries and tendinopathies. In several studies, PRP improved clinical outcome, increased tendon vascularity, decreased the retear rate, and relieved pain [14–18], whereas other studies showed that there was no benefit from PRP injection for tendon rupture or tendinopathies [19–24].

The aim of this randomized controlled trial (RCT) was to compare postoperative changes in plain radiographs and MRI in the periarticular structures after THA using the Hardinge approach with either PRP or placebo injection into the tendon area during wound closure. The hypothesis was that PRP improves the healing of the gluteus muscles (medius and minimus) and decreases the amount of gluteus muscle tears, atrophy, and fatty atrophy.

## Methods

### Study design

We performed a multicenter, double-blinded, randomized clinical trial (RCT) with parallel study groups. The ethics committee of the Hospital District of Southwest Finland and the local institutional research board approved the study.

### Study patients

Patients were recruited and operated on in three hospitals: Päijät Häme Central Hospital (Lahti, Finland), Turku University Hospital (Turku, Finland), and Satakunta Central Hospital (Pori, Finland).

A total of 40 patients were recruited from surgery queues. Before the preoperative visit, patients received a written invitation to participate in the study. During the visit, they received detailed information about THA treatment and the study and were given 2 weeks to consider participating in the study. Informed consent was obtained from each participant included in the study. Patients had the right to withdraw from the study at any stage. Patients were randomized into two groups: 19 in the PRP group and 21 in the placebo (PB) group. They underwent surgery between December 2015 and October 2020.

The ethics committee of the Hospital District of Southwest Finland approved the study (ETMK 77/2015, 21.3.2017).

### Inclusion and exclusion criteria

Patients were eligible for the study if they were aged 60–76 years, had moderate to severe radiographic hip osteoarthritis (Kellgren and Lawrence grade 3–4, [25]), and had symptomatic hip osteoarthritis requiring treatment with THA. Patients were ineligible for the study if they had had previous surgery on the affected joint, post-traumatic arthritis, rheumatoid arthritis, gluteus medius deemed abnormal during surgery (i.e., ruptured or atrophied), malignancy, oral glucocorticosteroid medication, insulin-dependent diabetes, smoking, alcohol or drug abuse, or mental instability.

### Intervention and follow-up

All patients underwent THA with the modified Hardinge approach under spinal anesthesia. In this procedure, the gluteus minimus and anterior third of the gluteus medius were split and detached from their insertions on the greater trochanter of the femur. After the component placement, the gluteus medius tendon was reattached to the greater trochanter with non-absorbable sutures via bone tunnels. The surgeon evaluated the condition of the gluteus medius muscle/tendon unit during surgery. If the gluteus medius was torn and detached from the bone anteriorly, the patient was withdrawn from the study. During closure, PRP (leukocyte-poor GLO PRP) or PB (NaCl 0.9%) was injected into the gluteus medius tendon incision line and also targeted into the muscle–tendon interval. Local infiltration analgesia (LIA) was not used. The surgeon did not know to which group the participant belonged. A staff nurse opened a sealed envelope and a study physician prepared the PRP or PB outside the operation room before it was handed to the surgeon.

All patients followed the same postoperative protocol. Rehabilitation included full weight bearing as tolerated with crutches, free range of movement, and physiotherapy instructions. A plain radiograph of the hip was taken postoperatively. Postoperative pain medication included paracetamol and opiates.

Postoperative visits were scheduled at 3 and 12 months after the operation. Postoperative radiological visits consisted of plain radiographs and MRI at the same time points. Orthopedic surgeons performed the clinical examination (limping, wound healing, movements of the hip joint).

### Postoperative imaging

The MRI scan took place 3 and 12 months after THA. Postoperative radiological visits were performed at three different hospitals. Three different MRI devices were used. The MRI device used in most scans was a Philips Healthcare Evolution 1.5T (Best, The Netherlands) with torso coil, which includes the FlexCoverage Anterior coil and FlexCoverage Posterior coil with up to 32 channels. Metal artifact reduction was applied in all sequences to diminish artifact formation from adjacent prosthetic hardware. The MRI sequences used in the study are shown in Table 4. Standard safety measures were applied before scanning.

T1-weighted sequences were used, first to evaluate the periprosthetic bony structures for any THA-related complications such as osteolytic changes or aseptic loosening; second, to examine any periprosthetic fluid collections or masses and suspected pseudotumors; and third, to identify any fatty degeneration and atrophy of the gluteal muscles. The latter was assessed using the Goutallier classification [26]. T2-weighted sequences were used to evaluate possible fluid collections and/or pseudotumors and the condition of gluteus medius and minimus tendon insertions on the greater trochanter of the femur. Short tau inversion recovery (STIR) sequences were mainly used to evaluate possible fluid collections related to the hip joint or surrounding structures or stress reactions of the bony structures.

In the current study, in which THA was performed using metal-on-polyethylene implants, the term pseudotumor is applied to describe fluid collections or solid masses connected to the hip joint and bulging outside the joint capsule. Pseudotumors meeting these criteria were graded using the Hart classification [27] based on their appearance in both T1 and T2-weighted MRI sequences and overall shape and capsule thickness morphology. Pseudotumor size was measured in three perpendicular dimensions.

One radiologist specialized in musculoskeletal radiology (JN) with 9 years’ experience analyzed all MR images three times and consulted another specialist in musculoskeletal radiology if a second opinion was needed.

Postoperative plain film radiographs were taken immediately after THA and 3 and 12 months after the operation. The first postoperative radiographs were taken in the supine position (anterior-posterior (AP) and horizontal beam lateral projections) and at later controls with AP projection in the standing position. Radiographs were analyzed for prosthesis positioning and any immediate or delayed complications such as intraoperative iatrogenic fractures, non-infectious osteolysis, or heterotopic ossification (HO). The Brooker classification was used to assess any bony formation between the greater trochanter of the femur and superior acetabular rim [28]. HO was evaluated with plain radiography and MRI. Plain radiographs were analyzed by the operating orthopedist and a musculoskeletal radiologist (JN)

### Platelet-rich plasma preparation

PRP samples were prepared by drawing 50 ml of venous blood from the patient’s antebrachial vein with 5 ml sodium citrate (anticoagulant). The samples were separated into 4 × 10 ml PRP syringes (GLO PRP kitTM, Glotech Co, Korea). PRP samples were separated using two separate centrifugations, the first for 5 min at 1200 rpm and the second for 2 min at 600 rpm. After the first spin, the red blood cells were collected and discarded. The second centrifugation was used to concentrate the platelets and separate the buffy coat from the PRP. Next, 2.5 ml of concentrated PRP was collected from the bottom of each syringe. From the final 10 ml PRP sample, a 1-ml sample was taken for laboratory analysis. The PB was sterile saline.

### Statistical analyses

The analyses were performed with JMP Pro 13.0 for Mac (SAS Institute Inc., Cary, NC). A *p*-value of <0.05 was considered to indicate statistical significance. The Shapiro-Wilks test (*p* >.05) was used to assess normality assumptions. Student’s *t*-test was used to compare the groups with normally distributed data and Wilcoxon’s rank-sum test was used for non-normally distributed data. Nominal data were tested using chi-square analysis. *p* <.05 (two-tailed) was considered statistically significant. Descriptive statistics (means, medians, standard deviations, and interquartile ranges) were used to describe the subject characteristics and results for the groups. Student’s *t*-test was used for normally distributed continuous clinical parameters; otherwise, the Mann-Whitney *U*-test was used. The results are expressed as mean values with standard deviations (SD) and as medians with interquartile ranges (IQR) when the normality assumption was not met.

## Results

Forty patients participated in this study. The mean age was 70 years (range 63–76). Twenty-three women (57.5%) and 17 men (42.5%) were included. There were no significant differences in patient characteristics between the PRP and PB groups. The patient demographics are shown in Table 1. One patient in the PB group could not be followed up with an MRI at 12 months postoperatively. All the follow-up X-rays were taken as planned. Uncemented femoral stems were used in 19 (47.5%) cases and uncemented cups in 35 (87.5%) cases. Cemented femoral stems were used in 21 (52.5%) and cemented cups in five (12.5%) cases. (Depuy Synthes, Raynham, MA, USA and Zimmer Biomet, Warsaw, IN, USA).

**Table 1:** Basic characteristics of patients in the study

Characteristic	PRP *n* = 19	Placebo *n* = 21	*p*-value	Total = 40
Age, mean (SD)	69.1 (4.2)	70.2 (3.9)	0.41	69.6 (4.0)
Sex				
M	7 (37%)	10 (48%)		17
F	12 (63%)	11 (52%)		23
BMI, mean (SD)	28.0 (4.1)	26.9 (4.3)	0.40	27.4 (4.2)
Atrial fibrillation	3 (15.8.%)	0	0.10	3
Coronary artery disease	1 (5.3%)	2 (9.5%)	0.87	3
High blood pressure	10 (52.6%)	8 (38.1%)	0.53	18
Hypercholesterolemia	4 (21.1%)	5 (23.8%)	0.72	9
Hypothyroidism	2 (10.5%)	1 (4.8%)	0.60	3
Type 2 DM	2 (10.5%)	2 (9.5%)	0.73	4
Insulin dependent	0	0		0

*SD* standard deviation, *BMI* body mass index, *DM* diabetes mellitus

**Fig. 1:**
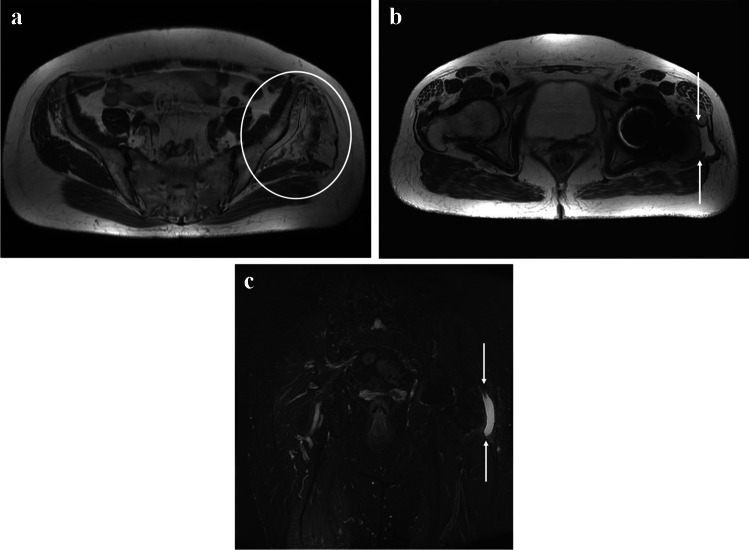
MRI showing left-side gluteus medius tendon tear at the level of insertion. Fatty atrophy on MRI **a** T1-weighted axial, **b** T2-weighted axial, and **c** STIR coronal

MRI findings of the gluteal muscles (gluteus medius and minimus) are shown in Tables 2 and 3. Gluteal muscle tear was identified in 11 patients (28%) at 3 and 12 months. The median length of the tear was 16 mm at 3 months postoperatively and 11 mm at 12 months. Gluteal muscle tear was found in four patients (21%) in the PRP group and seven patients (33%) in the PB group at 3 and 12 months. There was no significant difference in gluteal muscle tear between the PRP and the PB groups at 3 (*p*=0.38) and 12 months (*p*=0.33). No significant difference in gluteal muscle tear was found between 3 and 12 months. Figure 1 shows a gluteus medius tendon tear in postoperative MRI.

**Table 2:** Radiographic measurements of the gluteal muscles 3 months postoperatively

Radiographic measurements	PRP (*N* = 19)	PB (*N* = 21)	*p*-Value	Total (*N*=40)
Gluteal muscle tear (*n*/%)	4/21	7/33	0.38	11/28
Tear AP length (mm)^a^	14 (8–26)	17 (8–24)	0.82	16 (8–25)
Gluteal muscle atrophy (*n*/%)	10/53	6/29	0.15	16/40
Gluteal muscle fatty atrophy (*n*/%)	19/100	21/100	0.11	40/100
Normal muscle (Goutallier 0)	0	0		0
Some fatty streaks (Goutallier 1)	0	0		0
Less fat than muscle (Goutallier 2)	11	18		29/73
Amount of muscle is equal to fatty infiltration (Goutallier 3)	7	2		9/23
More fat than muscle (Goutallier 4)	1	1		2/5
Fluid collection (*n*/%)	8/42	10/48	0.72	18/45
HTO Brooker 1–4 (*n*/%)	6/32	3/14	0.19	9/23
Brooker 1	6/32	3/14		9/23
Brooker 2	0	0		0
Brooker 3	0	0		0
Brooker 4	0	0		0

*AP* anterior-posterior

^a^Data presented as median and interquartile range

**Table 3:** Radiographic measurements of the gluteal muscles 12 months postoperatively

Radiographic measurements	PRP (*N* = 19)	PB (*N* = 20)	*p-value*	Total (*N*=39)
Gluteal muscle tear (*n*/%)	4/21	7/35	0.33	11/28
Tear AP length (mm)^a^	14 (6–18)	10 (7–15)	0.91	11(7–16)
Gluteal muscle atrophy (*n*/%)	10/53	6/30	0.15	16/40
Gluteal muscle fatty atrophy (*n*/%)	19/100	21/100	0.086	40/100
Normal muscle (Goutallier 0)	0	0		0
Some fatty streaks (Goutallier 1)	0	0		0
Less fat than muscle (Goutallier 2)	10	17		27/69
Amount of muscle is equal to fatty infiltration (Goutallier 3)	7	2		9/23
More fat than muscle (Goutallier 4)	2	1		3/8
Fluid collection (*n*/%)	6/32	7/35	0.82	13/33
HTO Brooker 1–4 (*n*/%)	6/32	6/29	0.84	12/31
Brooker 1	4/21	5/25		9/23
Brooker 2	2/11	0		3/8
Brooker 3	0	1/5		1/3
Brooker 4	0	0		0

*AP* anterior-posterior

^a^Data presented as median and interquartile range

**Fig. 2:**
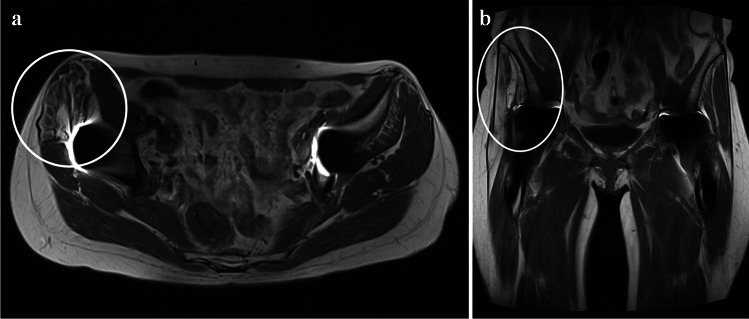
MRI showing right-side gluteus medius atrophy and Goutallier 4 fatty degeneration; T1-weighted sequences in **a** the axial and in **b** the coronal plane

Gluteal atrophy was seen on MRI in 16 patients (40%) at 3 and 12 months: 10 patients (53%) in the PRP group and six (29%) in the PB group (Table 4). No significant difference in gluteal atrophy was found between the PRP and PB groups at 3 (*p*=0.15) and 12 (*p*=0.15) months. Figure 2 shows gluteus medius atrophy in postoperative MRI. Fluid collection was found in 18 patients (45%) at 3 months and 13 patients (33%) at 12 months. In the PRP group, eight patients (42%) had a fluid collection at 3 months and six patients (32%) at 12 months. In the PB group, 10 patients (48%) had fluid collections at 3 months and seven patients (35%) at 12 months. There was no significant difference in the prevalence of fluid collections between the groups at 3 and 12 months postoperatively (*p*=0.72, *p*=0.82). No prosthetic joint infections were reported.

**Table 4:** MRI sequences used in the study

MRI sequence	Orientation	Anatomic region	TE (ms)	TR (ms)
T1 MARS	Coronal	Pelvis	16	455
STIR MARS	Axial	Pelvis	40	8570
T1 MARS	Axial	Unilateral hip	16	515
T2 MARS	Sagittal	Unilateral hip	80	4861
STIR MARS	Coronal	Unilateral hip	40	9021

*MRI* magnetic resonance imaging, *STIR* short tau inversion recovery, *MARS* metal artifact reduction sequence, *TE* time to echo, *TR* repetition time

**Fig. 3:**
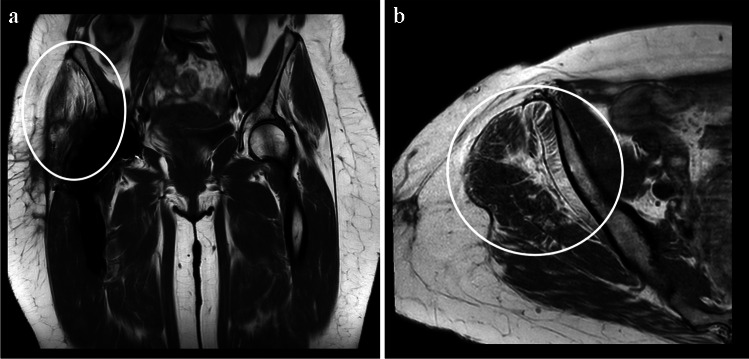
MRI showing fatty atrophy of the right-side gluteus minimus and medius. T1-weighted sequences in **a** the coronal and **b** the axial plane 3 months after THA. The findings indicate Goutallier 3 fatty atrophy

Fatty atrophy of the gluteal muscles was divided into 5 grades following the Goutallier classification. Grade 0–1 fatty atrophy was not observed in any patient at 3 or 12 months postoperatively. Grade 2 was seen in 29 patients (73%) at 3 months and 27 patients (69%) at 12 months. Grade 3 was found in nine patients (23%) at 3 and 12 months. Grade 4 was found in two patients (5 %) at 3 months and three patients (8%) at 12 months. There was no significant difference in fatty atrophy between the PRP and PB groups at 3 and 12 months (*p*=0.11, *p*=0.086). Figure 3 shows fatty atrophy of gluteus medius and minimus in postoperative MRI.

**Fig. 4:**
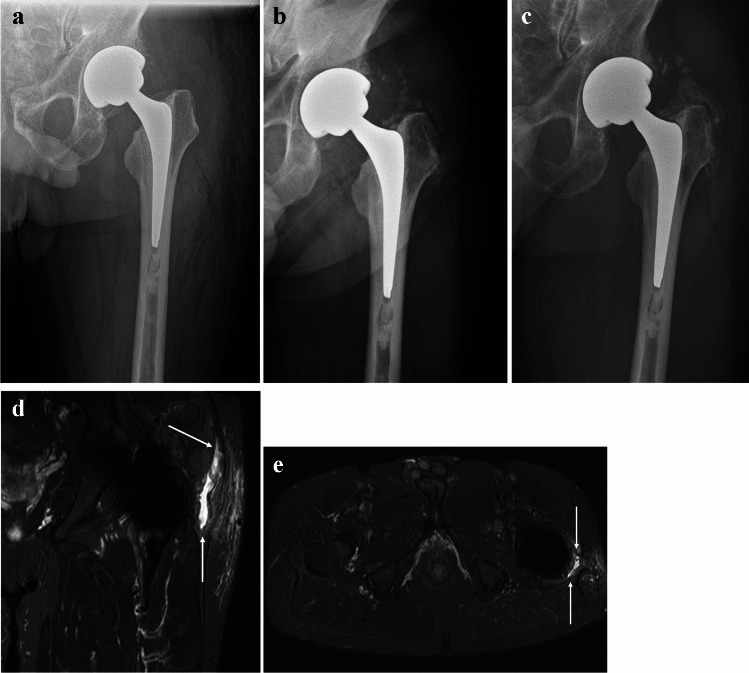
Heterotopic ossification development in a patient. AP radiograph **a** immediately postoperatively, **b** 3 months after THA presenting Brooker grade I, and **c** 12 months after THA presenting Brooker grade III. MRI of the same patient 3 months after THA, showing the active phase of calcified tendinitis in **d** the STIR coronal and **e** the STIR axial plane

After 3 months postoperatively, HO (Brooker I) was found in nine patients (23%). After 12 months, HO increased by one grade (Brooker I to II) in two patients (5%) and by two grades (Brooker I to III) in one patient (2.5%). After 12 months, three patients (7.5%) developed new HO (Brooker I). No significant difference was found in HO between groups at 3 and 12 months (*p*=0.19, *p*=0.84). PRP did not increase the formation of HO. Figure 4 shows development of HO in postoperative radiographs and MRI.

Three (7.5%) postoperative complications were reported: one (2.5%) temporary neuropraxia, one (2.5%) periprosthetic fracture, and one (2.5%) periprosthetic loosening. The periprosthetic fracture was noticed at the first operation and fixed with a cable system. The periprosthetic loosening needed revision arthroplasty after 9 months postoperatively. No prosthetic joint infections were reported.

## Discussion

We used MRI to evaluate gluteal muscle tears, atrophy, fatty atrophy, heterotopic ossification, and fluid collections in a randomized PRP vs PB trial after THA performed through the Hardinge approach. Fatty atrophy of gluteal muscles was a common finding after THA. PRP injection into the gluteus medius tendon did not improve healing detected by MRI.

In our study, gluteal tendon tear was detected in 28% of patients in both groups. Müller et al. reported similar results, with gluteus medius tendon defects in 37% of patients at 3 months and 25% at 12 months after THA. Gluteus minimus tendon defect was more common, reported in 62% and 50% of patients at 3 and 12 months, respectively [29]. Pfirrmann et al. reported gluteus minimus tendon defects in 38% of patients, lateral gluteus medius tendon defects in 44% of patients, and posterior gluteus medius tendon defects in 11% of patients after THA [11].

In our study, MRI showed fatty atrophy of the gluteus medius and minimus in all patients. No improvement was seen between 3 and 12 months. Previous studies have shown that muscle fatty atrophy is irreversible, even after successful tendon reconstruction [30, 31]. De Anta-Diaz et al. reported fatty atrophy of the gluteus medius in 51% of patients and of the gluteus minimus in 73.4% of patients after THA through a lateral approach at 6 months [32]. They used a dichotomized Goutallier classification, where only grades 2 to 4 were reported as fatty atrophy (moderate or severe). Mild fatty atrophy was classified as normal, which may explain the lower incidence of fatty atrophy compared to our study. In addition, their patients were younger than ours (63.5 years vs 70 years). Müller et al. showed that patients aged over 70 years had a higher degree of fatty atrophy of the gluteus medius compared to younger patients after THA [33]. In this study, there was no difference in preoperatively measured fatty atrophy between the groups. In previous studies, the correlation between gluteal atrophy and clinical outcome has shown conflicting results. One explanation could be the heterogeneity of classification used [34]. On the other hand, Pfirrmann et al. reported that fatty atrophy of the gluteus medius has been shown to correlate with clinical outcome [11].

There is no official classification system for fatty atrophy of the gluteus medius. The Goutallier classification is a grading system for fatty atrophy of the rotator cuff muscles of the shoulder [26]. Previous studies have also used a modified Goutallier classification for fatty atrophy of the hip abductor muscles after THA [11, 29]. Engelken et al. developed a new classification system (Quartile) for gluteal fatty atrophy and compared it to the Goutallier classification system. Both systems were equally accurate for predicting gluteal fatty atrophy after THA [35]. Klemet et al. showed that the Quartile classification system was superior to the Goutallier system in THA patients [36].

HO is a common finding after THA. The most common classification for HO is Brooker’s classification, which uses antero-posterior radiographs of the hip to divide HO formation into four grades [28]. Grades III and IV indicate clinically significant HO reducing the hip function [37]. The incidence of HO after THA is widely reported to be from 5 to 68% and clinically relevant HO to be from 1 to 9% [38, 39]. In our study, the incidence of HO was 30% at the 12-month follow-up, and only 2.5% of patients had clinically relevant HO (all grade III). Similar results have been reported in other studies. Zhu et al. reported that the incidence of HO after THA was 30% [40]. Wei et al. reported an HO incidence of 24% and Toom et al. of 32% after THA. In our study, PRP did not increase the formation of HO after THA. Early ossification can be diagnosed with plain radiography or computerized tomography (CT) [41]. On MRI, early and active ossification is seen as an edematous soft tissue reaction with findings of calcified tendinitis. At this stage, small ossification foci may easily be missed. Mature HO resembles cancellous bone with a cortical bone layer and fat marrow inside, which is also evident on the MRI. In our study, HO was evaluated with MRI and plain radiography. MRI is not required to evaluate HO, but it can help in assessing soft-tissue structures in case of active calcified tendinitis and also around matured HO.

MRI is often done when a pseudotumor is suspected. Langton et al. identified no clear consensus in the literature defining the boundaries of the terms metallosis, aseptic lymphocyte-dominated vasculitis-associated lesions (ALVAL), and pseudotumors. They used the term adverse reactions for metal debris to describe arthroplasty failures associated with pain, large sterile effusion, macroscopic necrosis, or metallosis [42]. Pseudotumors have been reported to develop in patients who have received conventional metal-on-polyethylene bearing hip replacements [42]. More recently, pseudotumors have been reported in relation to metal-on-metal bearing hip replacements [43–46]. In our study, no pseudotumors were reported.

Fluid collections were a common finding in our study; all were found around the greater trochanter and connected to the hip joint. Pfirrmann et al. reported that fluid collections were found in asymptomatic and symptomatic patients after THA but that collections of over 4 ml were seen only in symptomatic patients [11]. Müller et al. reported that fluid collections were found in 31% of patients at 3-month follow-up and in 12.5% of patients at 12-month follow-up after THA through the modified Hardinge approach [29]. In our study, fluid collections did not indicate infections either clinically or radiologically.

There are only a few studies on how PRP affects the healing process after THA. Capion et al. injected leukocyte platelet-rich plasma (L-PRP) onto the sutured fascia and found that it promoted wound healing after THA [47]. In a retrospective analysis, PRP reduced postoperative bleeding in THA [48]. Another retrospective analysis showed that using PRP in THA did not reduce postoperative bleeding or analgesic requirements or shorten the hospital stay [49]. We did not find any adverse events caused by PRP.

A limitation of our study was that we did not have any pre-operative MRI imaging. Osteoarthritis and aging increase the risk of gluteal fatty atrophy and tears [50, 51]. However, some studies have shown that only a few patients have gluteus medius or minimus fatty atrophy on MRI before THA, and no preoperative gluteus medius or minimus tendon tears were reported [29, 33]. De Anta-Diaz et al. reported that 2% of patients have moderate or severe fatty atrophy in the gluteus medius and 9% in the gluteus minimus on MRI before THA [32]. Due to these relatively low previously published atrophy/tear rates, we think that our post-operative MRI findings are of considerable interest even though pre-operative imaging was lacking. Another limitation of the study was that only one musculoskeletal radiologist evaluated the MR images systematically. Unfortunately, the other specialist involved in the study left their position at our hospital and was unable to double-check all the MR scans systematically. A further limitation concerns the relatively small sample size. The COVID-19 pandemic significantly slowed our recruitment and we decided to stop recruiting at that point. However, we still think that our results are of considerable interest with the current numbers. Further research on the subject is of course recommended.

The strength of the study was the prospective, randomized multicenter study design. MRI with very high sensitivity and specificity was used to assess tendon pathology up to 1 year postoperatively. Further, this is the first RCT we are aware of to evaluate the effectiveness of PRP in gluteal muscle healing after THA through the Hardinge approach.

We found that MRI is a valuable tool to evaluate postoperative tissue changes. Fatty atrophy of gluteal muscles was a common finding. PRP did not improve radiological healing and cannot be recommended for routine use.

## Data Availability

The data that support the findings of this study is available on request from the corresponding author. The data are not publicly available due to privacy or ethical restrictions.
